# Erdheim Chester disease in a patient with Burkitt lymphoma: a case report and review of literature

**DOI:** 10.1186/s13000-018-0772-2

**Published:** 2018-11-24

**Authors:** Hany I. Sakr, Kaila Buckley, Robert Baiocchi, Weiqiang John Zhao, Jessica A. Hemminger

**Affiliations:** 1 Department of Pathology, The Ohio State University Wexner Medical Center, 410 W. 10th Ave, N#308, Columbus, OH 43210 USA; 2 Department of Internal Medicine (Hematology), The Ohio State University Wexner Medical Center, Columbus, OH USA

**Keywords:** Erdheim Chester disease, ECD, BRAF, Burkitt lymphoma, Histiocytosis, Lymphoproliferative disorders

## Abstract

**Background:**

Erdheim Chester disease (ECD) is a rare, non-Langerhans cell histiocytosis characterized by widespread tissue infiltration by CD68-positive, CD1a-negative foamy histiocytes. ECD can be difficult to identify, and diagnosis relies on the presence of histiocytes with certain histologic and immunophenotypic features in an appropriate clinical and radiologic setting. Clinical signs and symptoms are variable depending on which organ systems are involved. Most patients have at least skeletal involvement with bone pain as well as fatigue. Other common manifestations include diabetes insipidus, cardiac, periaortic, or retro-orbital infiltration/fibrosis, kidney impairment, xanthelasmas, among others.

**Case presentation:**

Herein, we describe a case of *BRAF*-mutation positive ECD in a patient with Burkitt lymphoma, and we review recent literature.

**Conclusion:**

Underlying *BRAF* and other MAPK pathway mutations are identified in approximately 50% of cases of ECD, which aids in diagnosis as well as enables novel targeted treatments. ECD patients have an increased risk of myeloid neoplasms; however, unlike other histiocytoses, an association with lymphoproliferative disorders has not been recognized.

## Background

Erdheim Chester disease (ECD) is a rare histiocytosis characterized by tissue infiltration by CD68-positive, CD1a-negative foamy histiocytes [1–4]. The disease mostly affects adults with a mean age of diagnosis in the fifth or sixth decade, though rare pediatric cases have been reported [5–8]. Of note, due to rarity and nonspecific clinical and biopsy findings, there is commonly a delay in diagnosis with a reported average time to diagnosis of 4.2 years [4]. There is a slight male predominance (M:F = 1.5 to 3:1).

The disease has a wide spectrum of manifestations depending on distribution and degree of tissue involvement [4]. It is uncommon for ECD to involve only one organ system; thus, ECD patients typically have multiple signs and symptoms related to infiltration of multiple organ systems. The most common presenting clinical manifestations include bone pain that is symmetrically present involving long bones and diabetes insipidus due to infiltration of skull base and pituitary, respectively. Other common manifestations include renal impairment due to encasement of kidneys and involvement of the retroperitoneal space, cardiovascular disease due to encasement of the aorta and/or pseudotumor of the right atrioventricular groove, various endocrine abnormalities such as hypogonadism and hypothyroidism, and a variety of neurologic signs and symptoms, including mild cognitive impairment, cerebellar ataxia, and peripheral neuropathy. Two other characteristic lesions of ECD include periorbital xanthelasmas and retro-orbital involvement with resultant exophthalmos. Lungs are commonly involved but generally asymptomatic [3–19]. Although less common, ECD involvement of other organ sites, such as adrenal glands, gastrointestinal tract, thyroid, testis, breast, liver, and pancreas, have been reported [4, 20–25]. Radiologic imaging studies are useful to identify which organ systems may be involved.

Histologic features of ECD include an atypical accumulation of histiocytes, which commonly have a foamy appearance due to cytoplasmic lipid vacuoles. Associated stromal fibrosis and admixed Touton giant cells are also frequently present. There can be admixed small, mature lymphocytes, neutrophils, eosinophils, and/or plasma cells. There is no emperipolesis, necrosis, nuclear atypia, or well-formed granulomas. The histiocytes in ECD are positive for CD68, CD163, and Factor XIIIa and negative for CD1a and langerin [1, 2, 4, 16, 19, 24]. S-100 expression is variable. An underlying *BRAF* mutation (most commonly V600E) is seen in approximately half of ECD cases. Mutations in other MAPK pathway genes, including *MAP2K1*, *NRAS*, *KRAS*, *ARAF*, and *PIK3CA*, have also been reported in ECD [4, 26].

Herein we describe a case of *BRAF*-mutation positive ECD in a 48 year old male who was diagnosed with ECD after experiencing increasing weakness and failure to thrive after completing treatment for Burkitt lymphoma.

## Case presentation

### Clinical history

A 48 year old male with a past medical history of pituitary abnormality with central diabetes insipidus and hypogonadotrophic hypogonadism for eight years treated with desmopressin (DDAVP) and testosterone. He presented with shortness of breath, exertional dyspnea, and a four month history of weight loss and drenching night sweats. Computed tomography scan of the chest revealed a large mediastinal mass, and positron emission topography-computed tomography (PET/CT) scan demonstrated extensive, 2-[^18^F] fluoro-2-deoxy-D-glucose (FDG)-avid mediastinal and abdominal lymphadenopathy. A subsequent mediastinal lymph node biopsy showed Burkitt lymphoma with t(8;14), and a staging bone marrow was negative for lymphoma. Burkitt lymphoma was staged as IVB. The patient received four cycles of R-CODOX/M/IVAC (Rituximab, Cyclophosphamide, Oncovin [Vincristine], Doxorubicin, Ifosfamide, Vepesid [etoposide], and Ara-C [Cytarabine] with methotrexate held due to pleural effusions and ifosfamide deleted in cycles 2 and 4 due to neurotoxicity. He also received involved field radiotherapy of 40 Gy in 20 fractions to residual mediastinal/subcarinal/pleural disease. During this time he was diagnosed with central hypothyroidism and began treatment with levothyroxine.

After completing the chemotherapeutic regimen, the patient experienced waxing and waning pain in the lower back and knees as well as bilateral leg weakness. Magnetic resonance imaging (MRI) scan of the knee showed infiltrative lesions within the distal femoral metaphysis/diaphysis and proximal tibial diaphysis; however, PET/CT scan was negative for PET-avid bone disease. A bone biopsy was suboptimal with nonspecific findings. Bilateral iliac crest biopsies revealed mildly hypercellular bone marrow with a small non-paratrabecular lymphohistiocytic aggregate. Over the next six months he showed progressive failure to thrive with new onset dysphagia. Neurologic and endocrine evaluations remained negative. MRI scans of the thoracic and lumbar spines showed progressive bone disease and extensive lung and pleural disease. The patient underwent bone marrow and pleural/lung biopsies.

### Biopsy findings

**Fig. 1:**
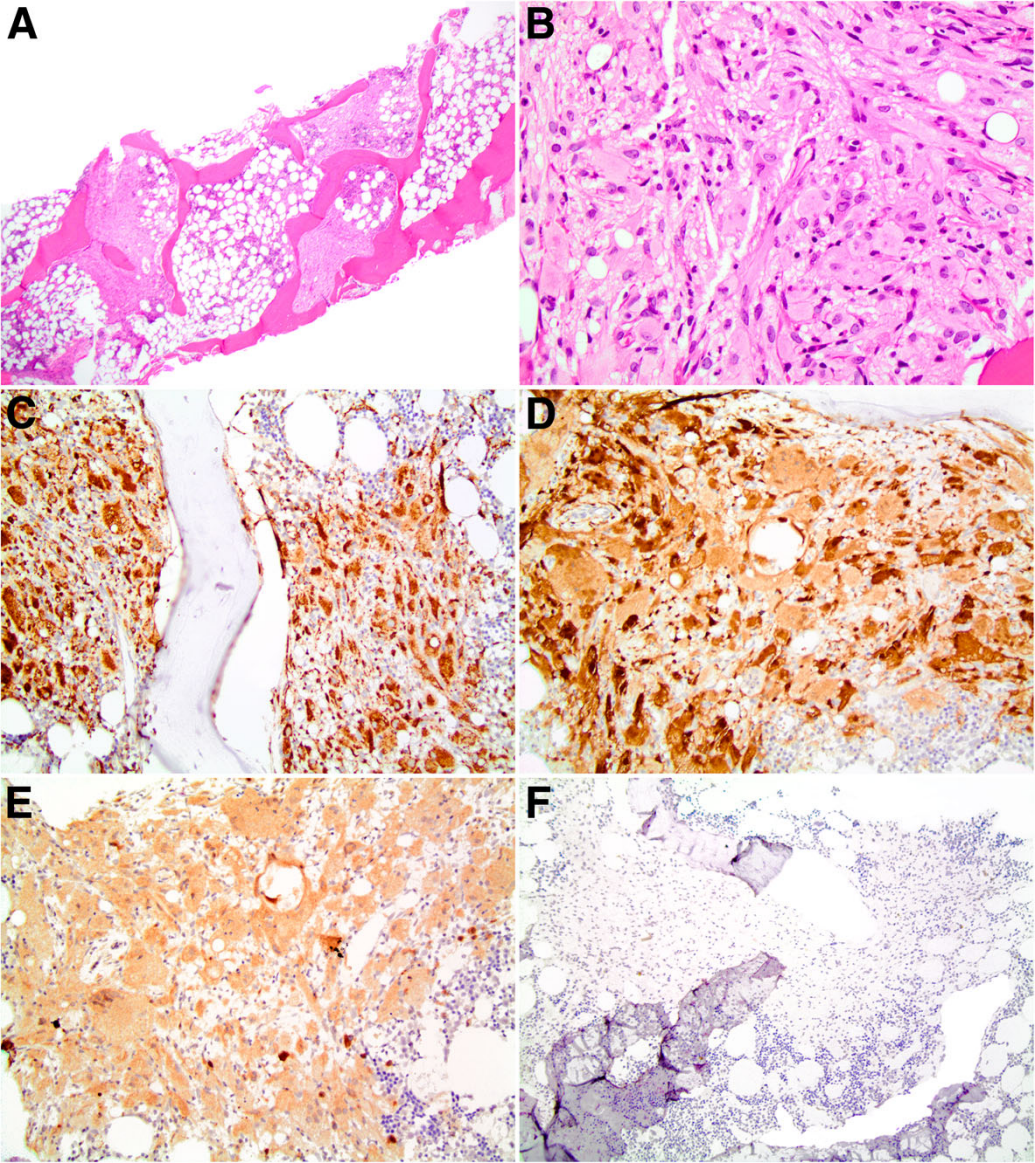
(**a**) Bone marrow biopsy shows cellular bone marrow with trilineage hematopoiesis and patchy, atypical histiocytic aggregates (H&E, 20x) (**b**) Atypical histiocytic aggregates are comprised of epithelioid to spindled-appearing histiocytes with a subset of foamy histiocytes (H&E, 400x). The atypical histiocytes are diffusely positive for (**c**) CD68 (200X) (**d**) Factor XIIIa (200x), and (**e**) BRAF ^V600E^ (clone VE1) and negative for (**f**) CD1a

The bone marrow biopsy showed normocellular bone marrow with preserved trilineage hematopoiesis and a few variably-sized, atypical histiocytic aggregates (Fig. 1). The histiocytes were epithelioid to spindled in appearance with a subset of foamy histiocytes. A rare multinucleated histiocyte was noted. The histiocytes were positive for CD68, CD163, factor XIIIa, and BRAF^V600E^ (clone VE1) and negative for CD1a, CD21, and CD23. There was no evidence of Burkitt lymphoma.

The pleural/lung lesion biopsy contained a small amount of tissue with a nonspecific fibrohistiocytic infiltrate. Genomic DNA was extracted and polymerase chain reaction (PCR) was performed for exon 15 of BRAF gene. The product was analyzed by pyrosequencing for mutations in codons 600 and 601 of BRAF with an analytic sensitivity of 5% for mutated allele. The *BRAF* p.V600E mutation (c.1799 T > A) was detected.

The histologic findings in combination with histiocyte phenotype, presence of *BRAF* mutation, and clinical and radiologic data supported a diagnosis of ECD.

## Discussion

ECD is a rare histiocytosis that was first described by William Chester in 1930 [27]. Histiocytoses are a heterogeneous group of diseases that historically have been classified as Langerhans cell or non-Langerhans cell subtypes with dendritic, macrophage/monocytic, and malignant forms [1]. However, recent insights have resulted in a proposed revised classification for histocytoses that includes five groups based on clinical, radiographic, pathologic, phenotypic, genetic, and/or molecular features [1]. In the proposed revised classification, Langerhans cell histiocytosis (LCH), ECD, and extracutaneous juvenile xanthogranuloma (JXG) are included in a group termed the “L group” since these entities share certain molecular and clinical features as well as can coexist in the same patient. In fact, nearly 20% of patients with ECD have concomitant LCH lesions [1, 19, 28, 29]. The “L group” histiocytoses commonly show clonal mutations causing constitutive activation of the MAPK pathway with *BRAF* mutations being the most common. The other four groups in the proposed revised classification are the “C group” (cutaneous non-LCH), the “R group” (Rosai Dorfman Disease [RDD] and variants), the “M group” (primary and secondary malignant histiocytoses), and the “H group” (hemophagocytic lymphohistiocytosis and macrophage activation syndrome).

Given the variable and nonspecific clinical presentation, a diagnosis of ECD can be delayed and diagnostic possibilities based on clinical findings include sarcoidosis, malignancy, infection, autoimmune disorder NOS, large vessel vasculitis, IgG4-related disease, pituitary adenoma, Paget’s disease of the bone, lysosomal storage disease, and multiple sclerosis [4]. Unfortunately, the histology is not specific either. The presence of foamy histiocytes with Touton giant cells is helpful in identifying ECD; however, additional immunophenotyping and molecular studies as well as clinical and radiologic correlation are necessary for a definitive diagnosis. The main histologic differential diagnosis includes nonspecific xanthogranulomatous inflammation, granulomatous inflammation associated with infections, non-histiocytic hematopoietic neoplasms, and other histiocytoses, including LCH, RDD, JXG, and histiocytic sarcoma. Immunophenotyping can be used to distinguish ECD from other diseases in the differential diagnosis (Table 1). Particularly, CD1a and langerin will be positive in LCH and negative in ECD. S-100 is typically strongly positive in RDD. Also, RDD is characterized by emperipolesis and negativity for Factor XIIIa. Since JXG and ECD are morphologically and immunophenotypically identical, their differentiation from one another mostly relies on the clinical presentation and radiologic findings. Cases of extracutaneous or widely-disseminated JXG are usually accepted as ECD [1]. ECD can typically be differentiated from malignant lesions since ECD lacks cytologic atypia, necrosis, and mitoses. Mycobacterial and fungal stains as well as cultures can be performed to exclude an infectious etiology. The lack of well-formed granulomas makes sarcoidosis less likely.

**Table 1:** Summary of immunohistochemical staining pattern for ECD differential diagnoses

	ECD	LCH	ICH	RDD	JXG
CD68	+	+	+/−	+	+
CD163	+	+	+/−	+	+
FXIIIa	+	–	–	–	+
CD1a	–	+	+	–	–
CD207 (Langerin)	–	+	–	–	–
S100	+/−	+	+	+	–
CD4	+	+	+	+	+
Lysozyme	+	+	+	+	+
BRAF^V600E^	+/−	+/−	+/−	–	+/−

*ECD* Erdheim Chester Disease, *ICH* Indeterminate cell histiocytosis, *JXG* Juvenile xanthogranuloma, *LCH* Langerhans Cell Histiocytosis, *RDD* Rosai-Dorfman Disease

Unlike RDD and JXG, which can have a benign clinical course and exhibit spontaneous regression, ECD progresses without treatment [4]. The traditional first line treatment for ECD is interferon alpha (IFN-α) or pegylated IFN-α [30]. A 2011 study by Haroche and colleagues showed that treatment with IFN-α or pegylated IFN-α was a major independent predictor of survival [31]. Unfortunately, IFN-a therapy is often poorly tolerated, however, a wide variety of other regimens have demonstrated disease activity, including cytokine-neutralizing antibodies, corticosteroids, chemotherapy, imatinib, cladribine, among others [4, 26, 32]. Recent studies utilizing BRAF and MEK inhibitors (e.g. vermurafenib, dabrafenib, trametinib) have shown efficacy in treating ECD with limited toxicity [29, 30, 33, 34].

The existence of both ECD and Burkitt lymphoma in this patient is interesting. It is recognized that histiocytic and dendritic cell neoplasms can be clonally related to leukemias and lymphomas, possibly due to derivation from a common pluripotent precursor, anaplastic progression with aberrant expression of some histiocyte/dendritic cell markers, or by “transdifferentiation” [1, 35, 36]. The typical scenario is a patient with a low grade B cell lymphoma such as follicular lymphoma and metachronous or synchronous histiocytic sarcoma or LCH. Clonal relationships have been proven at a molecular level by detecting the same immunoglobulin rearrangement and/or certain genetic abnormalities, such as t(14;18), in the histiocytosis and lymphoproliferative disorder [1, 35, 36]. Recently, Papo et al. reported an increased prevalence of myeloid neoplasms, specifically myeloproliferative neoplasms, myelodysplastic syndrome, and chronic myelomonocytic leukemia (CMML), in adults with non-Langerhans cell histiocytoses, including ECD alone or mixed histiocytosis (defined as ECD plus LCH or ECD plus LCH and RDD) [37]. In these patients the ECD commonly harbored a *BRAF*V600E mutation and the myeloid neoplasm commonly had *JAK2*V617F and/or other commonly mutated genes in myeloid neoplasms. In one patient, the same *NRAS* mutation was detected in both ECD and CMML.

Although there can be clonal relationships between certain histiocytoses and lymphoproliferative disorders and there is an increased risk of myeloid neoplasms in ECD patients, there is currently no known association between lymphoproliferative disorders and ECD. Other than our patient, there have only been a few reports of lymphoma in ECD patients. Papo et al. noted only two patients with lymphoma in their cohort of 189 cases of ECD/mixed histiocytosis. Also, there was a report describing marginal zone lymphoma in a patient with ECD [38]. Thus, lymphomas can occur in patients with ECD; however, a defined relationship has not been established, and the rare coexistence of ECD and lymphoma may be incidental.

## Conclusion

The diagnosis of ECD can be difficult and relies on clinical, histologic, phenotypic, radiologic, and molecular correlation. Clinical presentation is variable depending on organ system involvement; however, in patients presenting with bone pain and diabetes insipidus, the possibility of ECD should be considered, and imaging studies and biopsy may be useful. ECD histology is nonspecific, but characterized by an accumulation of foamy histiocytes with Touton giant cells. Unlike LCH, the histiocytes in ECD are Factor XIIIa positive and CD1a and langerin negative. Approximately 50% of ECD will have an underlying *BRAF* V600E mutation; thus, BRAF and MEK inhibitors are attractive therapeutic options. Lastly, ECD patients have an increased association with LCH as well as myeloid neoplasms.

## Abbreviations

CMML: Chronic myelomonocytic leukemia
DDAVP: Desmopressin
ECD: Erdheim Chester disease
IFN-α: Interferon alpha
JXG: Juvenile xangthogranuloma
LCH: Langerhans cell histiocytosis
PET/CT: Positron emission topography-computed tomography
R-CODOX/M/IVAC: Rituximab, Cyclophosphamide, Oncovin [Vincristine], Doxorubicin, Ifosfamide, Vepesid [etoposide], and Ara-C [Cytarabine]
RDD: Rosai Dorfman disease
